# Supplementary material for: Origins of lithium inventory reversibility with an alloying functional layer in anode-free lithium metal batteries
Source: Nat Commun. 2025 Aug 5;16:7216. doi: 10.1038/s41467-025-62289-6 (PMC12325613; doi:10.1038/s41467-025-62289-6)
Supplement: Supplementary file 1 — Supplementary Information [file 41467_2025_62289_MOESM1_ESM.pdf]

Supporting Information: Origins of lithium inventory reversibility with an alloying functional layer in anode-free lithium metal batteries

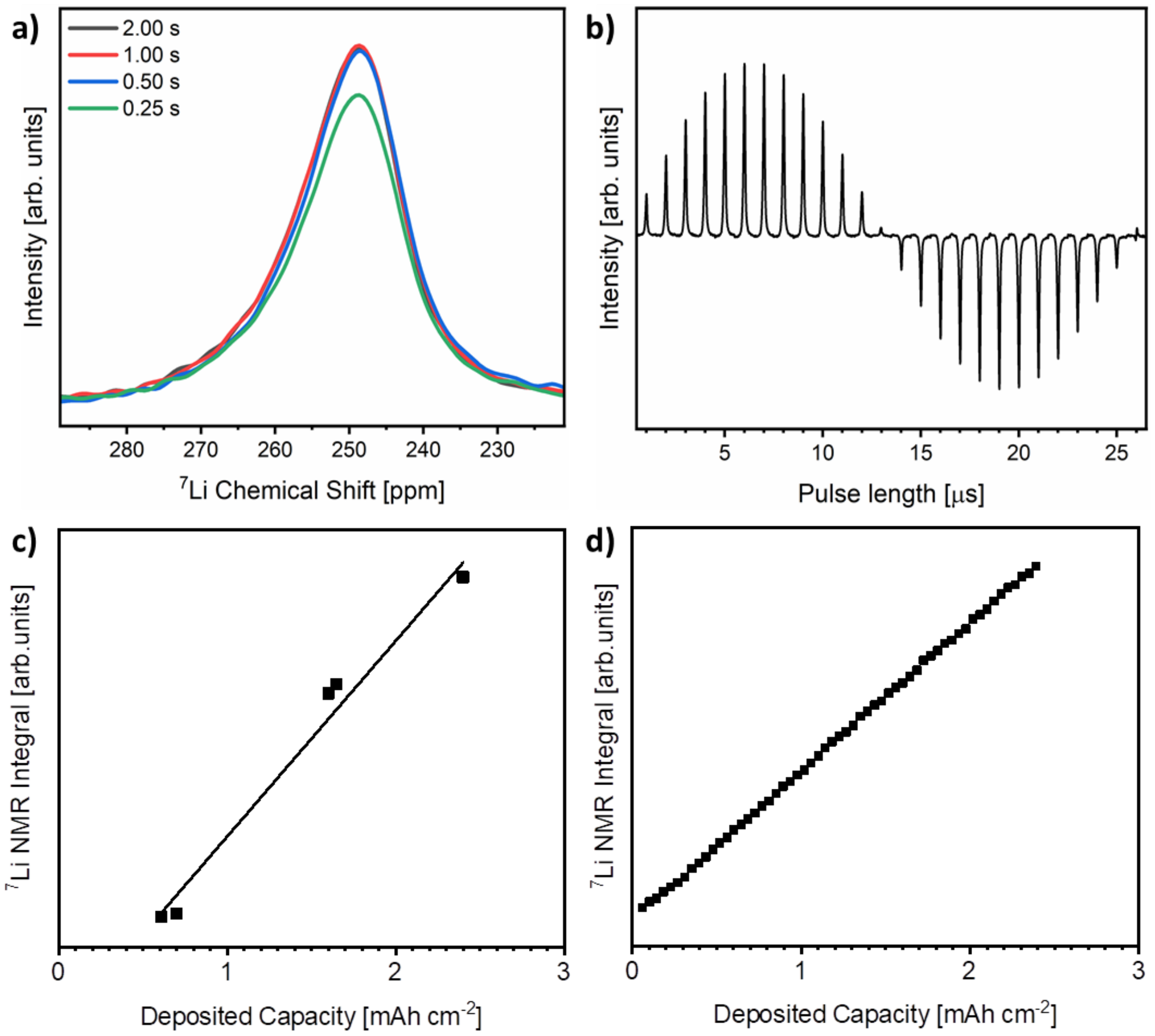

Supplementary Figure 1. **^{7}Li NMR detection of lithium metal deposits in Cu||NMC622 NMR pouch cells.** **a** Recycle delay at 100% SOC. **b** Pulse length at 100% SOC. **c** Linear relationship between deposited capacity and in situ ^{7}Li NMR intensity for multiple cells. **d** Linear relationship between deposited capacity and ^{7}Li NMR intensity for an operando cell. Electrolyte: LHCE (LiFSI:DME:TTE = 1:1.2:3 *n*:*n*:*n*).

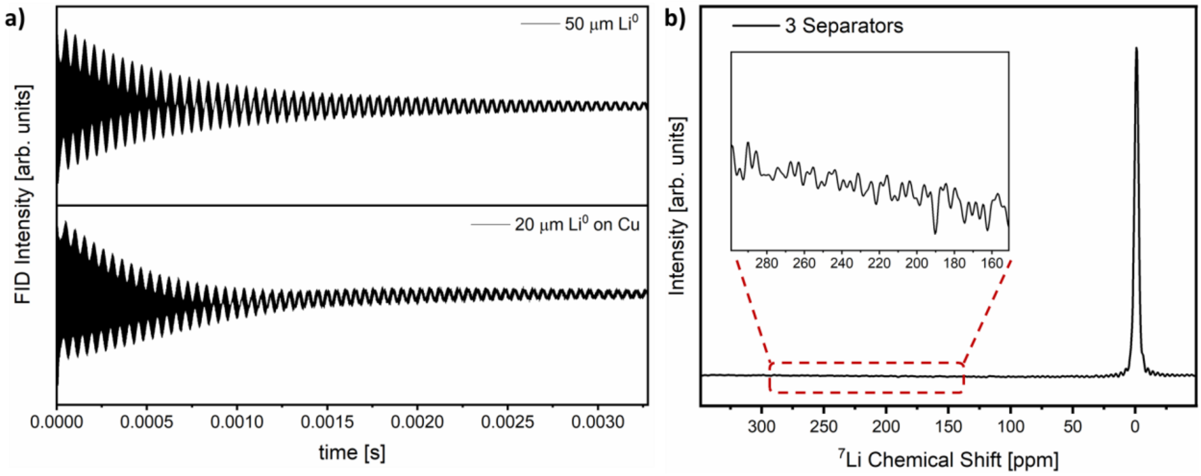

Supplementary Figure 2. **^{7}Li NMR experiments on separate cell components. a** Comparison of the FID-decay/signal attenuation for commercial lithium metal and lithium metal on copper electrodes sealed into NMR pouch foil. **b** ^{7}Li NMR spectra for three separators harvested from Cu||NMC622 NMR pouch cells utilized for calibration of the lithium metal integral and chemical shift (Supplementary Figure 3).

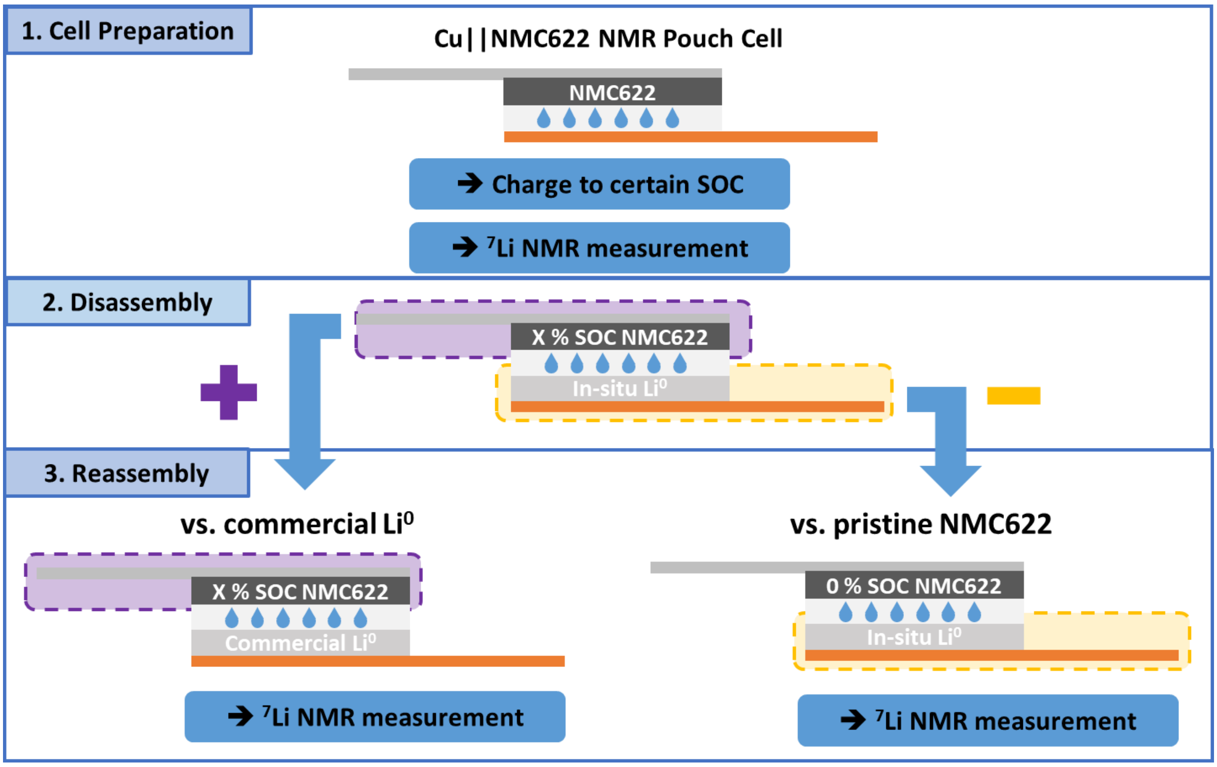

Supplementary Figure 3. **Experimental procedure for the calibration of positive electrode impact on NMR integrals and ^{7}Li chemical shifts of metallic lithium species.**

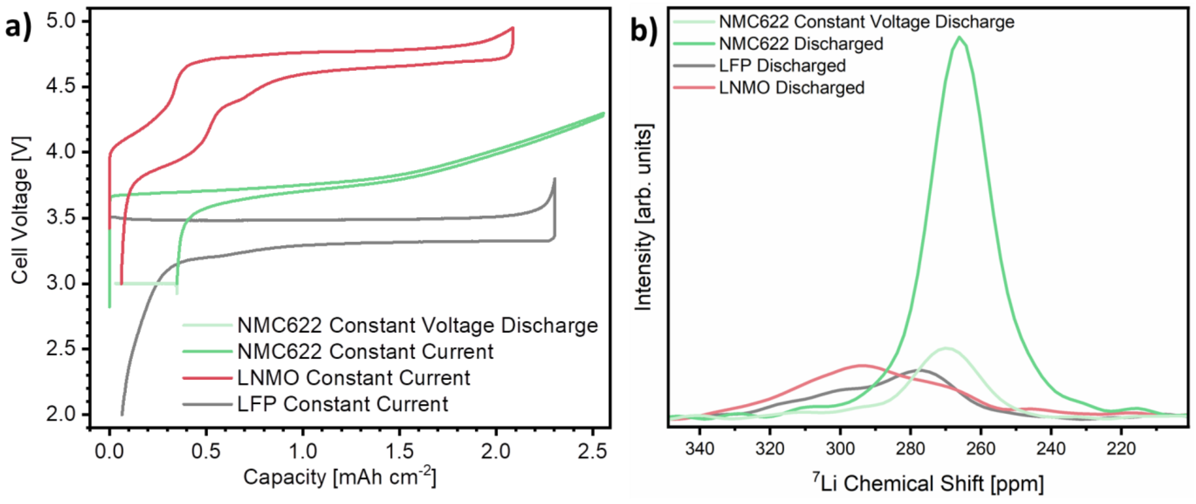

Supplementary Figure 4. **Electrochemical impact of selected positive electrodes.** **a** Voltage vs. capacity profiles for the initial cycle of lithium metal batteries operated with NMC622, LFP or LNMO positive electrodes. **b** Comparison of NMR spectra representing dead Li^0 for anode-free lithium metal batteries operated with the NMC622, LFP or LNMO positive electrodes. Electrolyte: 1 M $LiPF_6$ in EC/DEC (3:7 wt. by wt.) + 5 wt.% FEC.

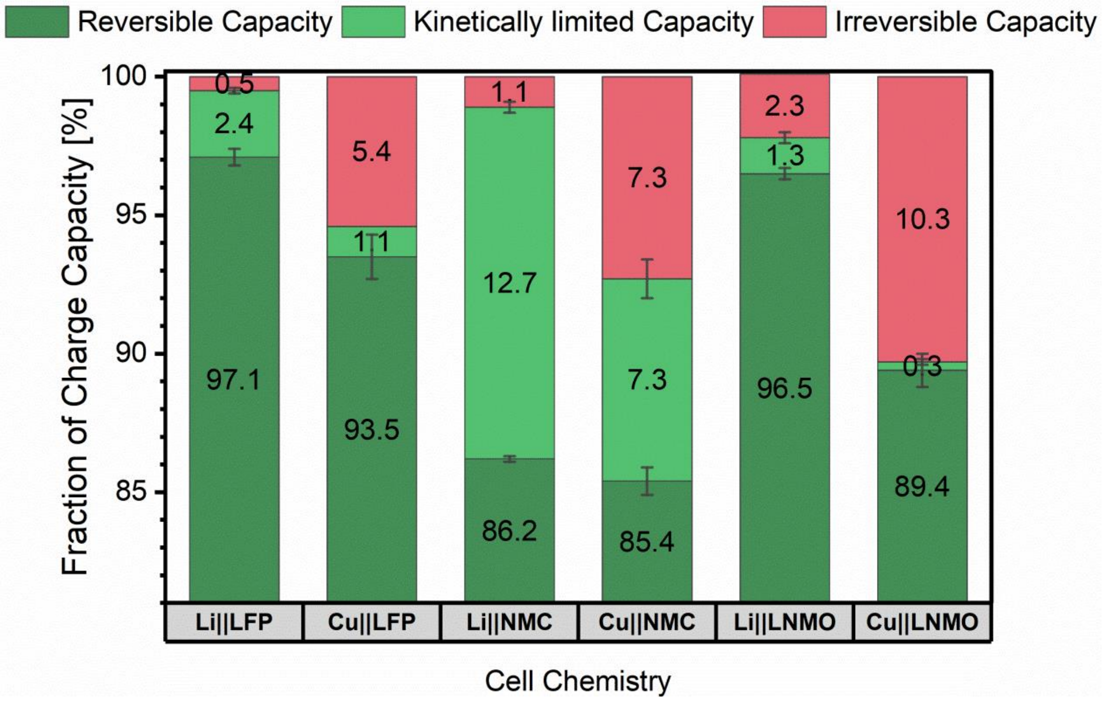

Supplementary Figure 5. **1st cycle capacity distribution in conventional and anode-free lithium metal batteries using LFP, NMC and LNMO as the positive electrodes.** Electrolyte: 1 M $LiPF_6$ in EC/DEC (3:7 wt. by wt.) + 5 wt.% FEC. Reversible capacity is determined by a constant current discharge at 0.6 mA cm^{-2}. Kinetically limited capacity refers to the capacity obtained in a constant voltage discharge at the lower cut-off voltage until reaching a current of less than 1 µA. Irreversible capacity is defined as the charge capacity that is not restored by constant current and constant voltage discharge. Error bars represent the standard deviation between at least two nominally identical cells.

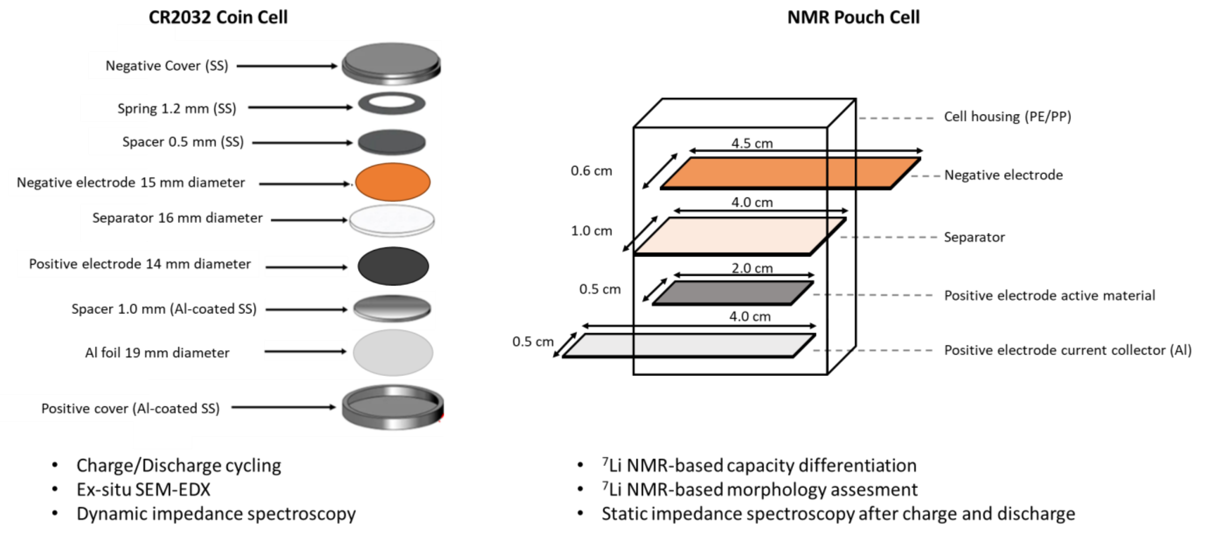

Supplementary Figure 6. **Overview of two electrode cell configurations and their utilization within this study.** Coin cell drawing adapted with permission from [1].

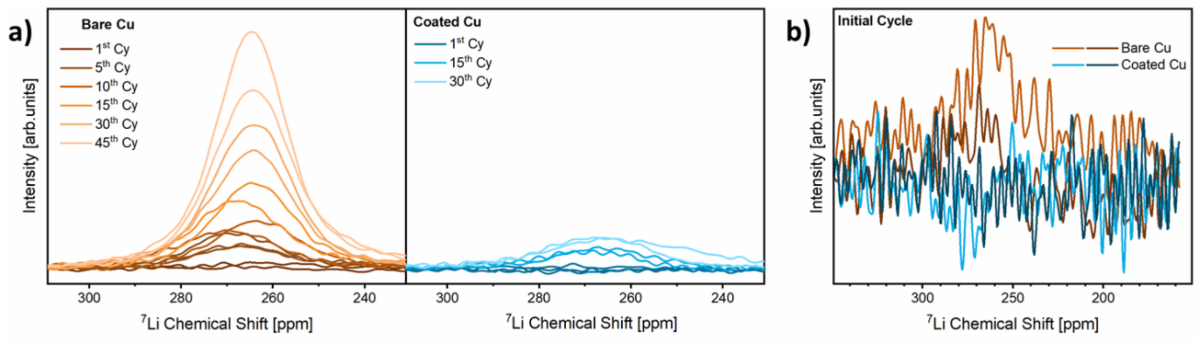

Supplementary Figure 7. **^{7}Li NMR spectra recorded after the CV step with only dead lithium remaining for Cu||NMC622 and coated Cu||NMC622 cells. a** Accumulation of dead lithium on bare and coated copper negative electrodes throughout cycling. **b** Close-up of the dead lithium deposits after the initial cycle. In a and b, multiple spectra are depicted for each cycle to demonstrate the reproducibility of experiments. Electrolyte: LHCE (LiFSI:DME:TTE = 1:1.2:3 *n*:*n*:*n*).

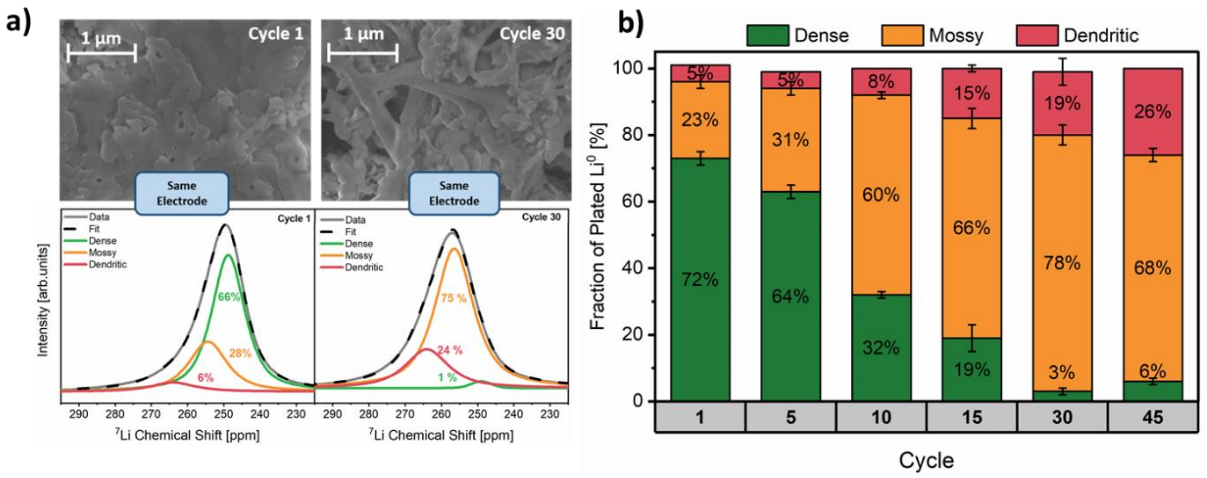

Supplementary Figure 8. **Lithium metal deposit morphology in Cu||NMC622 NMR pouch cells. a** Comparison of metallic lithium deposit morphology via SEM and ^{7}Li NMR. After operation, cells were first subjected to ^{7}Li NMR measurements and subsequently disassembled in a dryroom atmosphere to conduct SEM experiments with the identical electrode. **b** Evolution of the ^{7}Li NMR-based lithium deposit morphology on bare copper throughout cycling. (LiFSI:DME:TTE = 1:1.2:3 *n*:*n*:*n*). Error bars represent the standard deviation between at least two nominally identical cells.

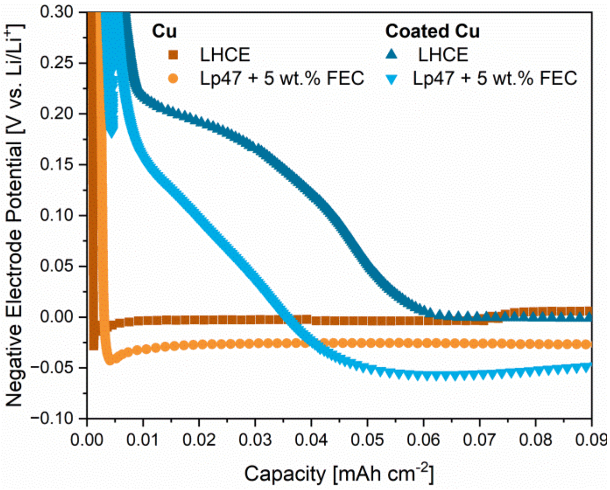

Supplementary Figure 9. **Nucleation overpotentials for lithium deposition in Cu||Li and coated Cu||Li three-electrode Swagelok cells.** Electrolyte: LHCE (LiFSI:DME:TTE = 1:1.2:3 *n*:*n*:*n*) or 1 M $LiPF_6$ in EC/DEC (3:7 wt. by wt., Lp47) + 5 wt.% FEC. Current density: 0.2 mA cm^{-2}.

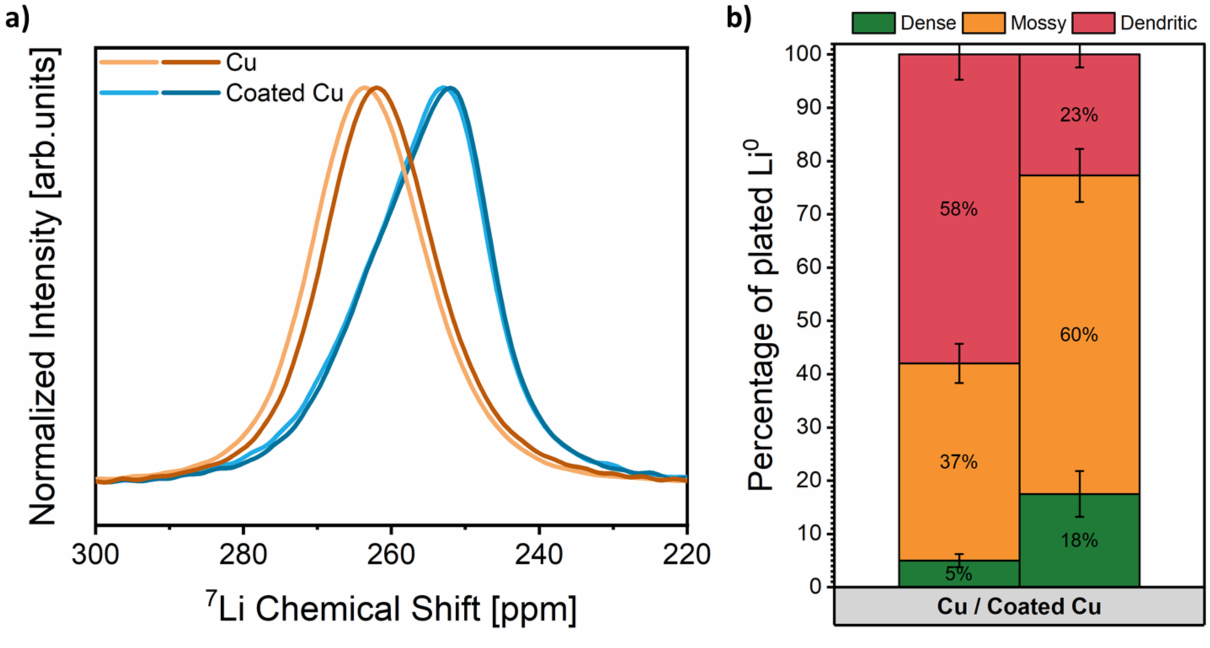

Supplementary Figure 10. **^{7}Li NMR-based lithium deposit morphology on bare and coated copper with a carbonate-based electrolyte.** **a** ^{7}Li NMR spectra with two datasets for each negative electrode to display their reproducibility. **b** Deconvolution of the 7Li NMR spectra displayed in a. Electrolyte: 1 M $LiPF_6$ in EC:DEC (3:7 wt. by wt.) + 5 wt.% FEC electrolyte. Current density: 0.2 mA cm^{-2}. Error bars represent the standard deviation between at least two nominally identical cells.

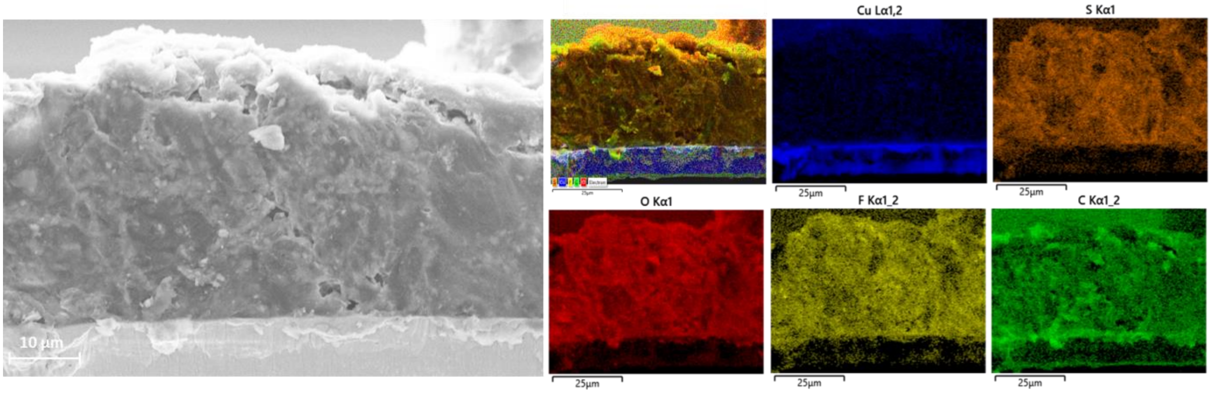

Supplementary Figure 11. **Cross-sectional SEM image and EDX mapping of the initial lithium deposition in Cu||NMC622 coin cells charged to 100% SOC.** Accelerating voltage: 10 kV. Electrolyte: (LiFSI:DME:TTE = 1:1.2:3 *n*:*n*:*n*).

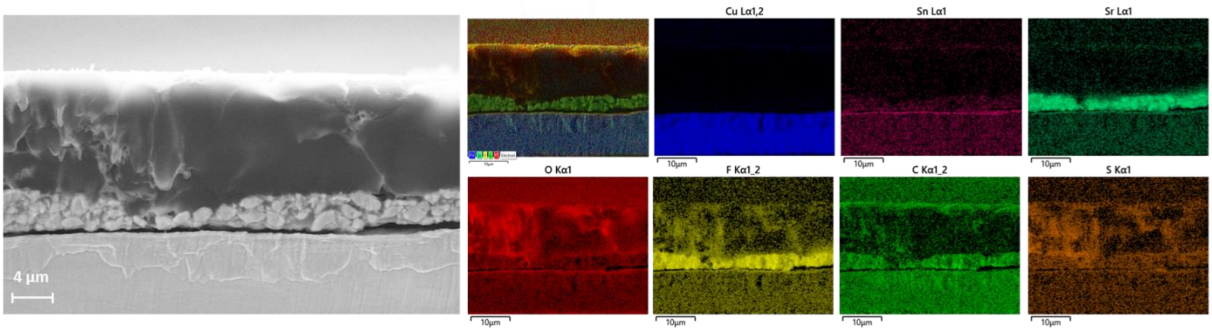

Supplementary Figure 12. **Cross-sectional SEM image and EDX mapping of the initial lithium deposition in coated Cu||NMC622 coin cells charged to 100% SOC.** Accelerating voltage: 10 kV. Electrolyte: LHCE (LiFSI:DME:TTE = 1:1.2:3 *n*:*n*:*n*).

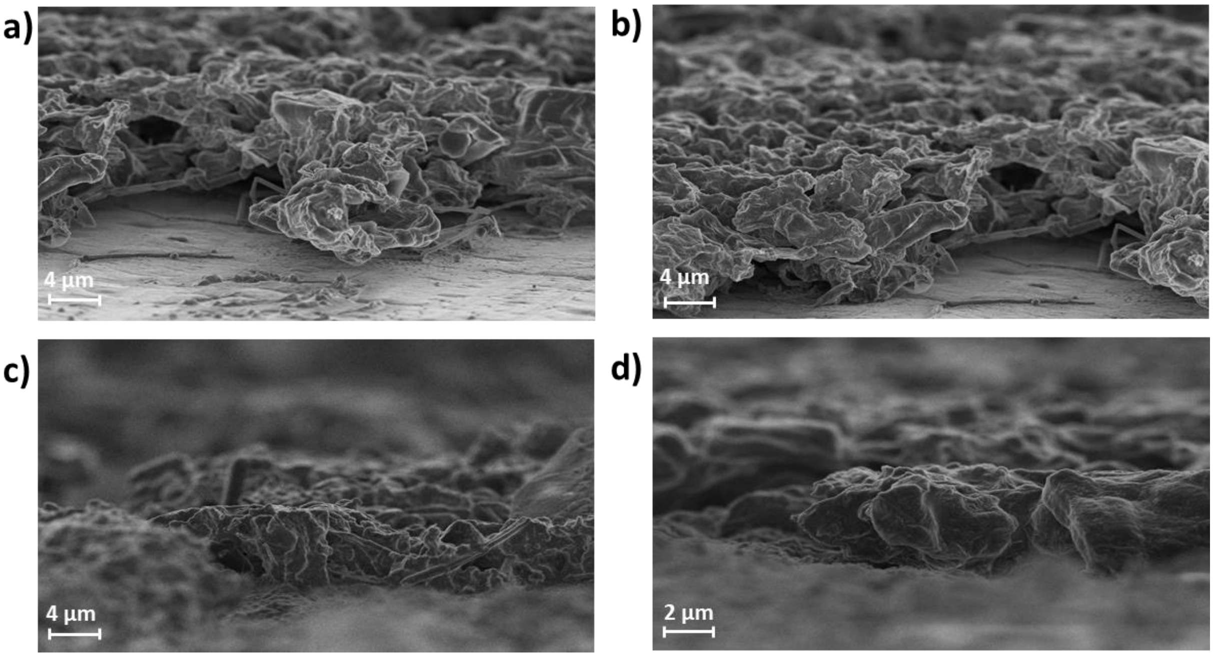

Supplementary Figure 13. **Cross-sectional SEM images after the initial discharge.** **a** and **b** Cu||NMC622 coin cells. **c** and **d** Coated Cu||NMC622 coin cells. Accelerating voltage: 3 kV. Electrolyte: LHCE (LiFSI:DME:TTE = 1:1.2:3 *n*:*n*:*n*).

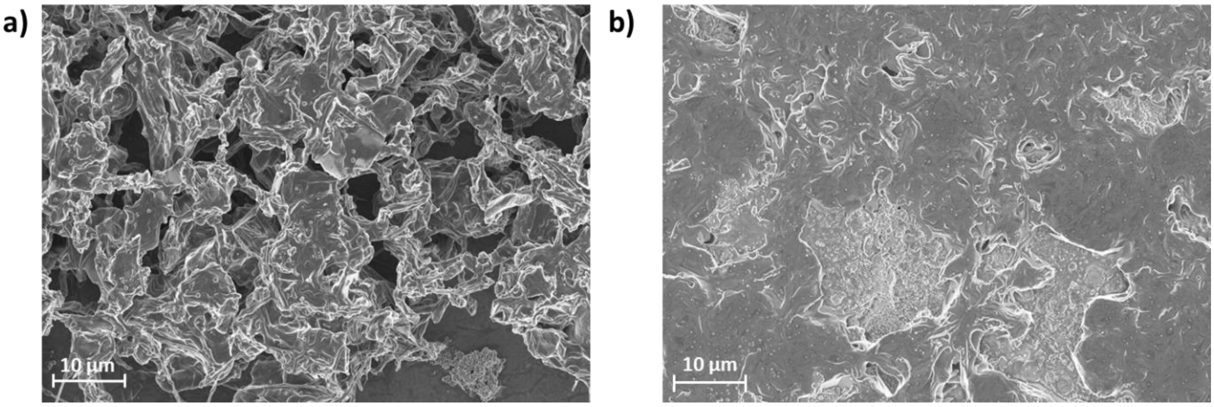

Supplementary Figure 14. **Top view SEM images after the initial discharge. a** Cu||NMC622 coin cells. **b** Coated Cu||NMC622 coin cells. Accelerating voltage: 3 kV.

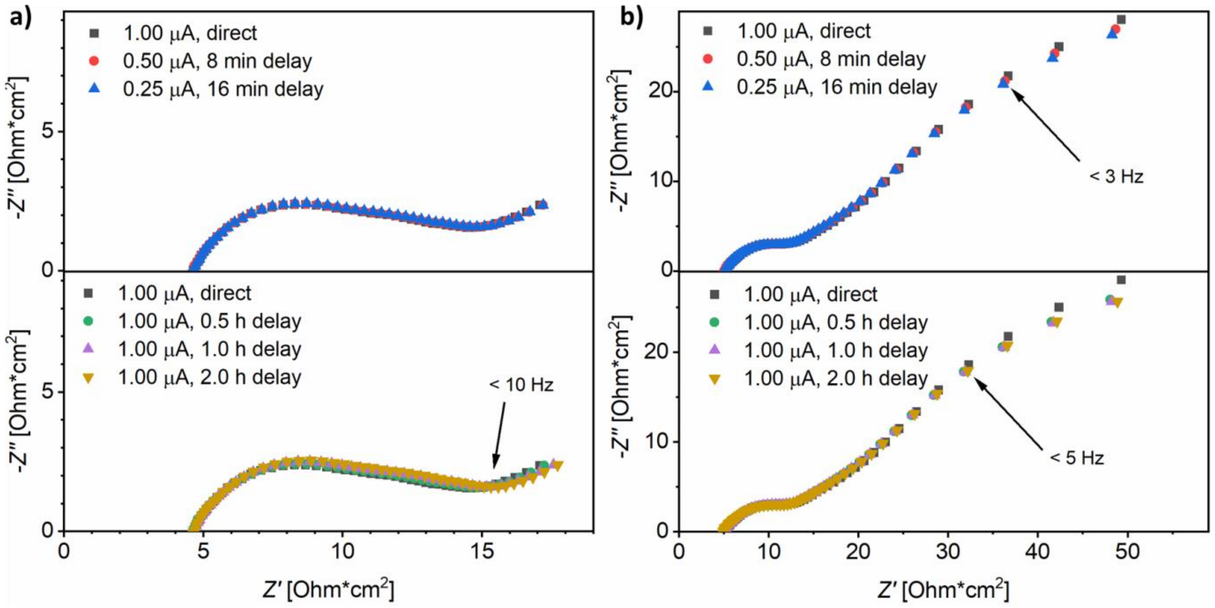

Supplementary Figure 15. **Linearity (top) and stability (bottom) tests for electrochemical impedance spectroscopy experiments with Cu||NMC622 NMR pouch cells. a** After initial charge **b** After initial discharge. The frequency for data points showing notable variation of impedance over time or amplitude is indicated. Electrolyte: LHCE (LiFSI:DME:TTE = 1:1.2:3 *n*:*n*:*n*).

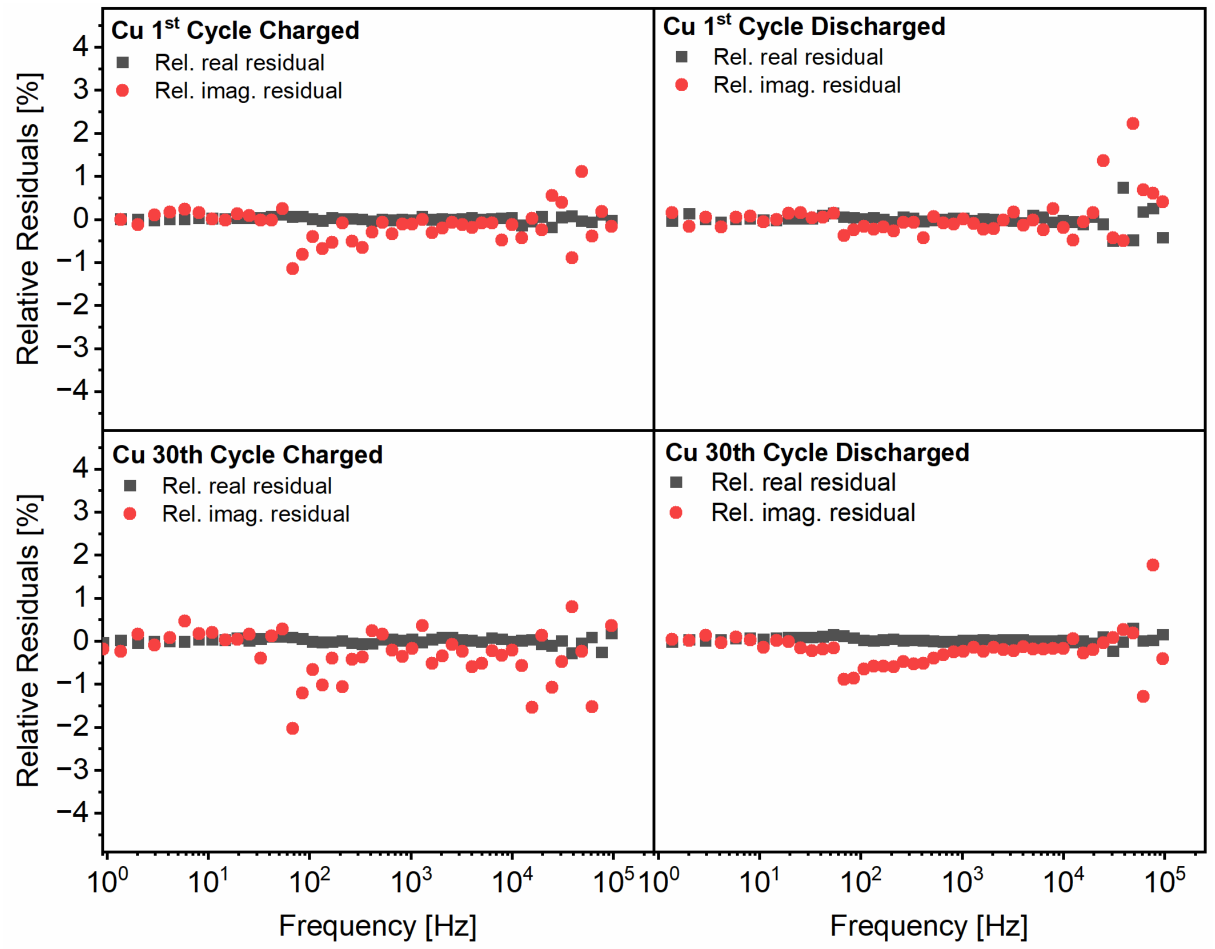

Supplementary Figure 16. **Kramers-Kronig tests for Cu||NMC622 NMR pouch cells in the charged and discharged state of the 1st and 30th cycle.** Frequency range: 100 kHz – 1.0 Hz. Electrolyte: LHCE (LiFSI:DME:TTE = 1:1.2:3 *n*:*n*:*n*).

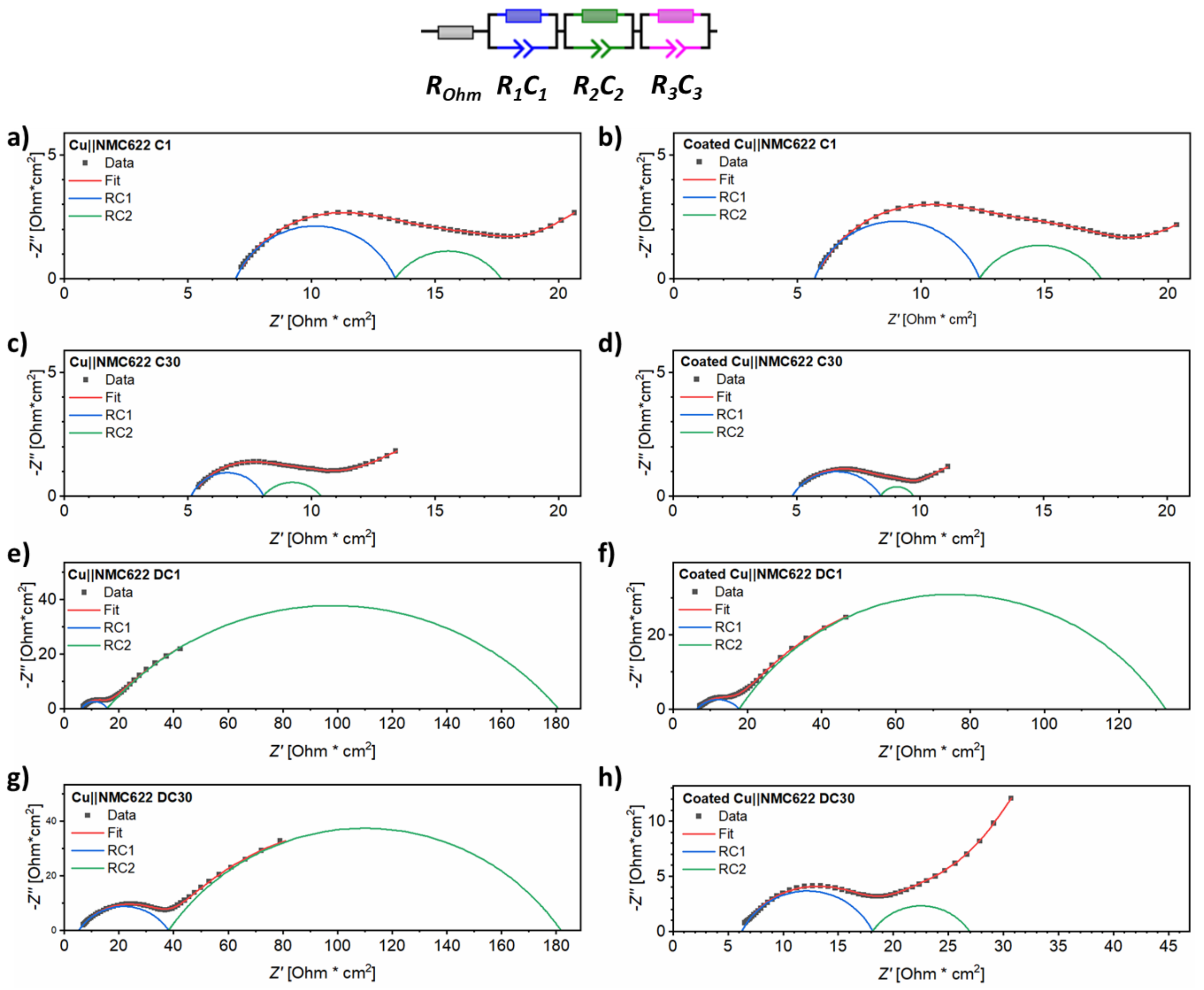

Supplementary Figure 17. **Equivalent circuit and fit of Nyquist plots of Cu||NMC622 and coated Cu||NMC622 NMR pouch cells in the charged and discharged state of the 1st and 30th cycle.** Frequency range: 100 kHz – 1.0 Hz. Electrolyte: LHCE (LiFSI:DME:TTE = 1:1.2:3 *n*:*n*:*n*).

Note that some equivalent circuit fits (i.e. **Supplementary Fig. 17e**, **f** and **g**) are of reasonable uncertainty, since the exclusion of data points in the high (> 100 kHz) and particularly low (< 1 Hz) frequency regime according to Kramers-Kronig tests renders some of the semi-circles incomplete. Furthermore, a third R-CPE element has been implemented to improve fitting of EIS spectra in which a third semi-circle occurs at the low frequency limit (i.e. **Supplementary Fig. 17a-d** and **h**). Due to very limited data points and scaling of the Nyquist plot, this R-CPE element is not visualized in the fit. In all cases, equivalent circuit fits are depicted as a visual guide for the distinction of processes, supporting the conclusions drawn in the main manuscript, where impedance spectra are discussed qualitatively rather than quantitatively.

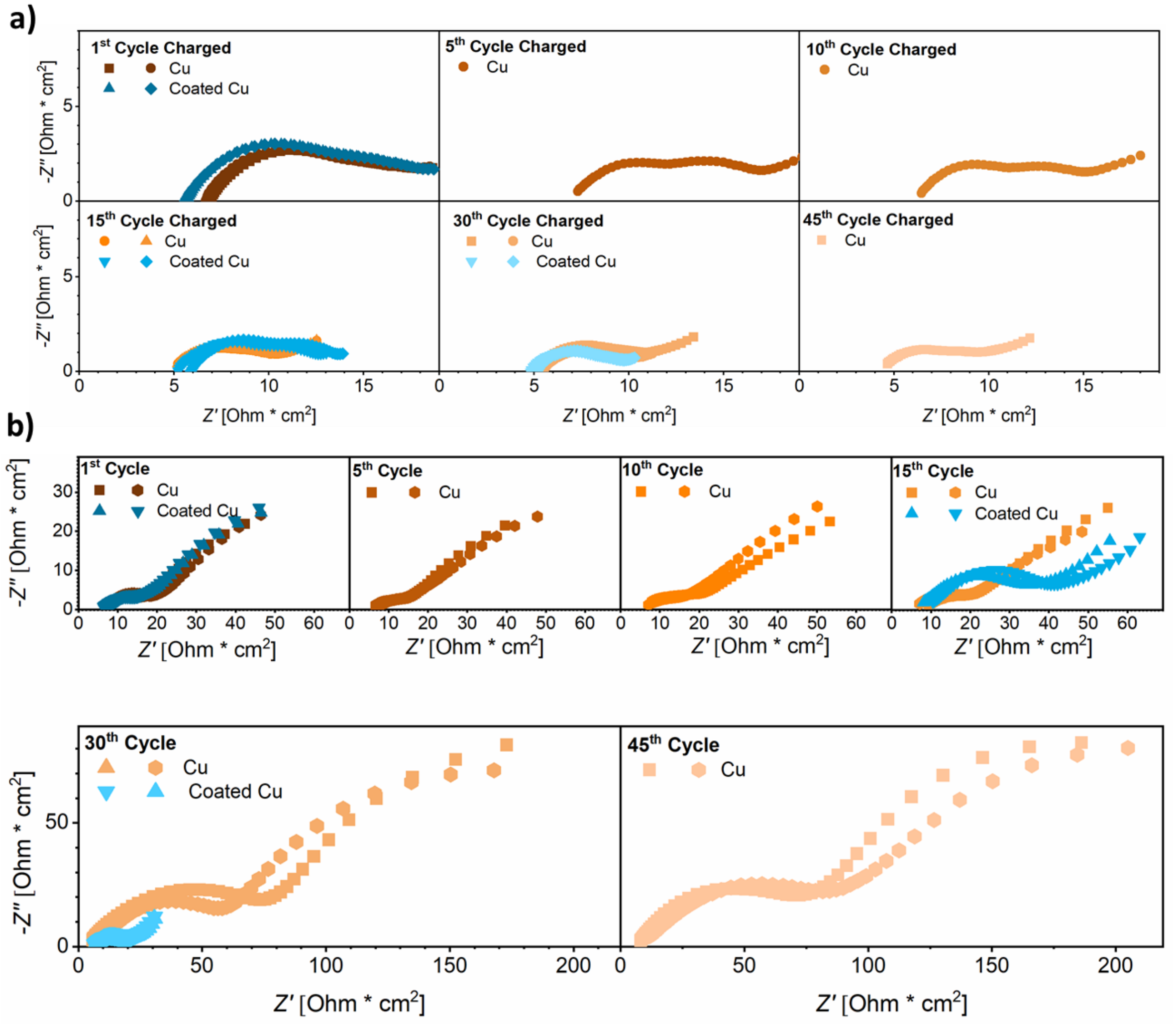

Supplementary Figure 18. **Nyquist plots of impedance spectra measured for Cu||NMC622 and coated Cu||NMC622 NMR pouch cells. a** After charge. **b** After discharge. When applicable, a second data set is depicted to display the reproducibility of impedance spectra. Frequency range: 100 kHz – 1.0 Hz. Electrolyte: LHCE (LiFSI:DME:TTE = 1:1.2:3 *n*:*n*:*n*).

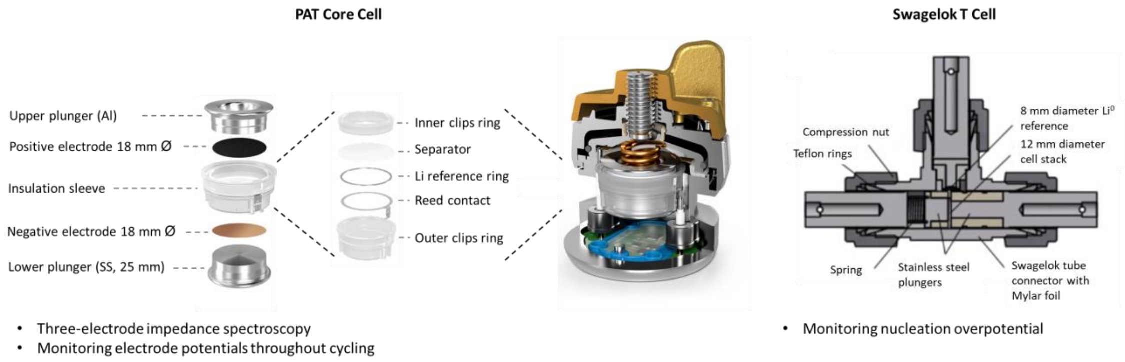

Supplementary Figure 19. **Overview of three-electrode cell configurations and their utilization within this study.** PAT Core Cell drawing adapted with permission from [2], Swagelok T Cell drawing adapted with permission from [3].

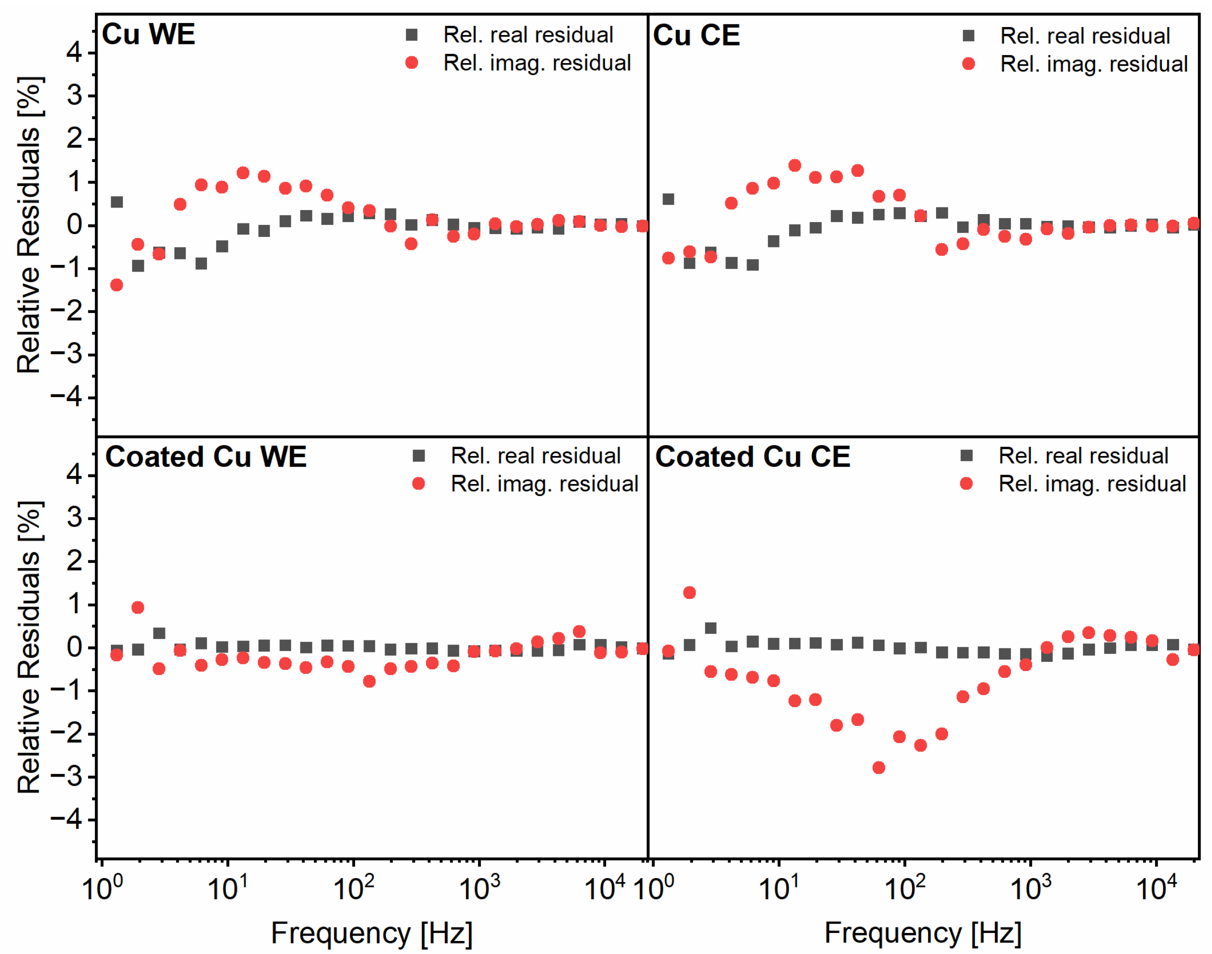

Supplementary Figure 20. **Kramers-Kronig tests for the impedance spectra of individual electrodes in Cu||NMC622 and coated Cu||NMC622 three-electrode PAT cells in the discharged state of the 15th cycle.** Frequency range: 20 kHz – 1.0 Hz. Electrolyte: LHCE (LiFSI:DME:TTE = 1:1.2:3 *n*:*n*:*n*).

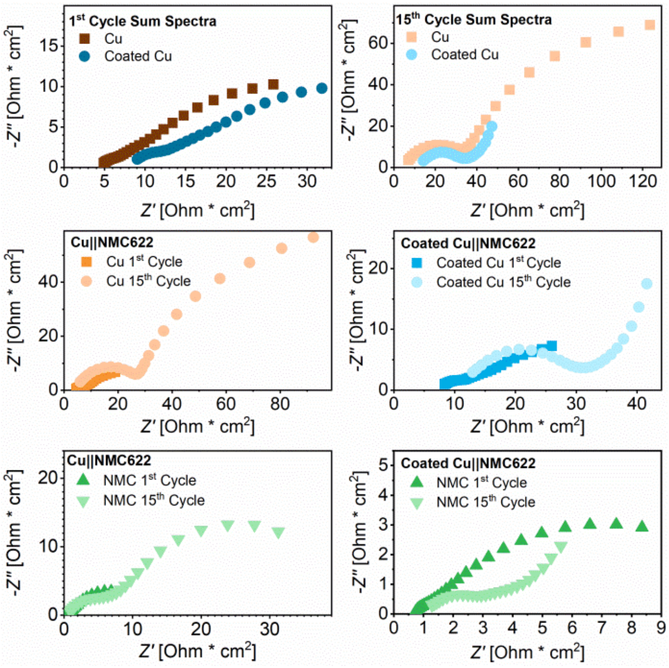

Supplementary Figure 21. **Representative summed (top) and individual electrode (middle, bottom) Nyquist plots for impedance spectra of three-electrode Cu||NMC622 and coated Cu||NMC622 PAT cells in the discharged state of the 1st and 15th cycle.** Frequency range: 20 kHz – 1.0 Hz. Electrolyte: LHCE (LiFSI:DME:TTE = 1:1.2:3 *n*:*n*:*n*).

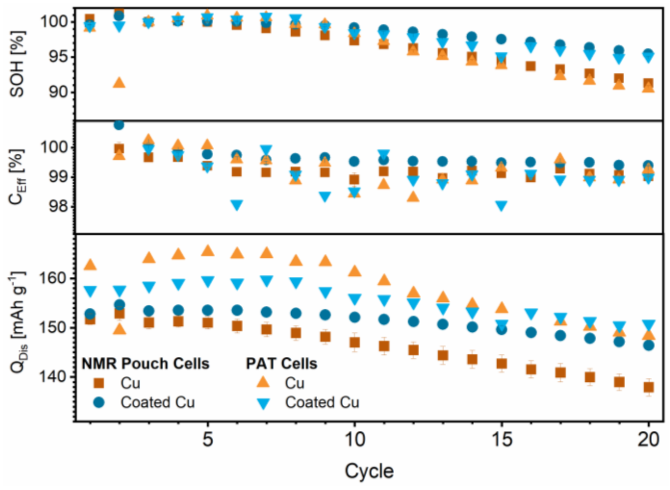

Supplementary Figure 22. **Comparison of electrochemical behavior of Cu||NMC622 and coated Cu||NMC622 cells in NMR pouch and PAT core cell format.** Electrolyte: LHCE (LiFSI:DME:TTE = 1:1.2:3 *n*:*n*:*n*). Error bars represent the standard deviation between at least two nominally identical cells.

For cells using bare and coated copper an accelerated capacity decay, i.e. reduced and less stable Coulombic efficiencies, are observed in three-electrode PAT cells. We suggest that this is due to pressure differences between the utilized cell formats, with increased pressure on NMR pouch cells resulting in improved reversibility of lithium inventory. Most likely, the earlier onset of increased cell resistance in the discharged state is due to an accelerated consumption of excess lithium in conjunction with the amplification of irreversible processes.

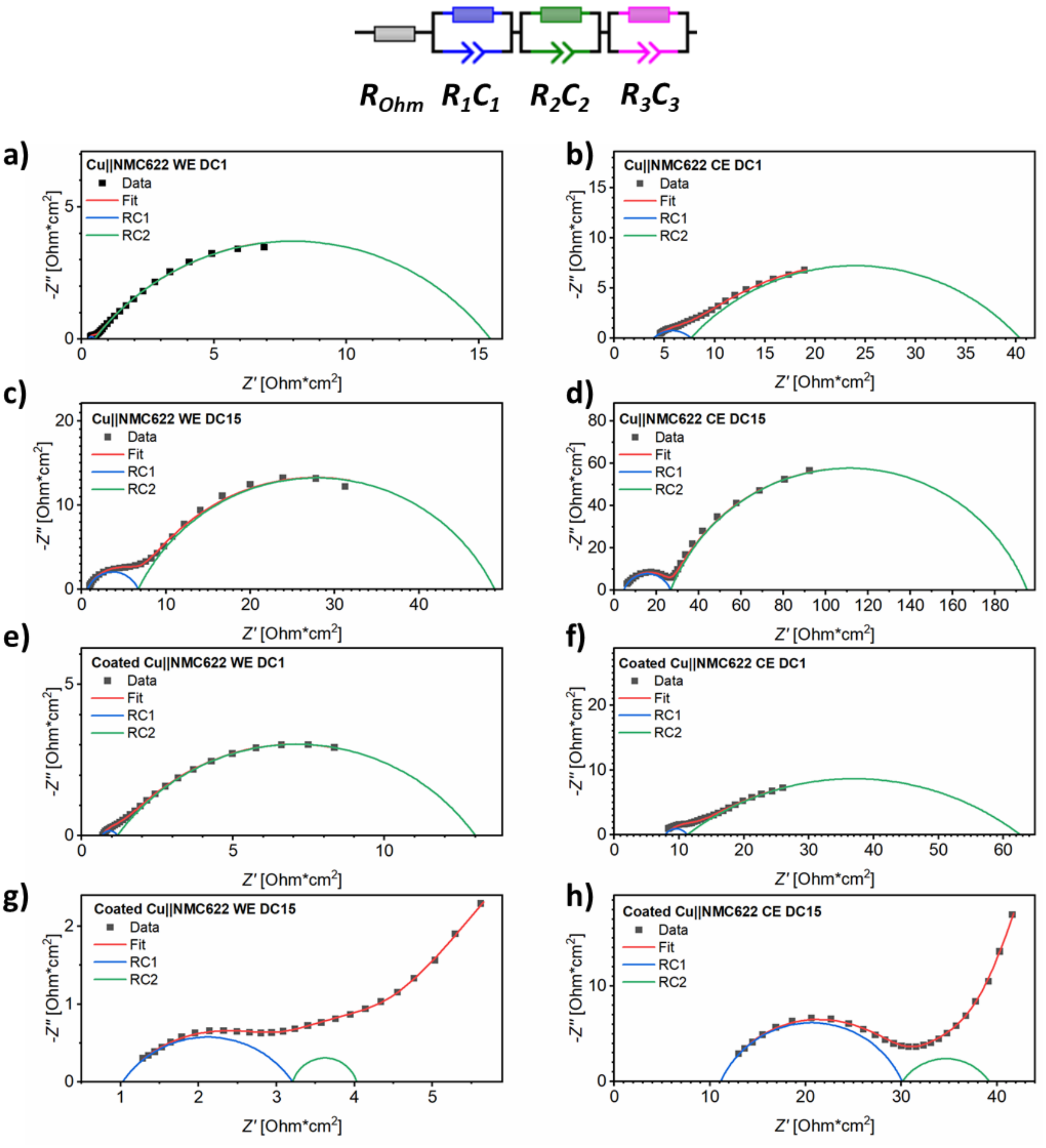

Supplementary Figure 23. **Equivalent circuit and fit of Nyquist plots for three-electrode Cu||NMC622 and coated Cu||NMC622 PAT cells in the discharged state of the 1st and 15th cycle.** The reference electrode allows differentiation of positive (working electrode, WE) and negative electrode (counter electrode, CE) impedance contributions. Frequency range: 20 kHz – 1.0 Hz. Electrolyte: LHCE (LiFSI:DME:TTE = 1:1.2:3 *n*:*n*:*n*).

Similar to **Supplementary Fig. 17,** most equivalent circuit fits (i.e. **Supplementary Fig. 23a-f**) are of reasonable uncertainty, since the exclusion of data points in the high (> 20 kHz) and particularly low (< 1 Hz) frequency regime according to Kramers-Kronig tests renders some of the semi-circles incomplete. Furthermore, a third R-CPE element has been implemented to improve fitting of EIS spectra in which a third semi-circle occurs at the low frequency limit (i.e. **Supplementary Fig. 23g** and **h**). Due to very limited data points and scaling of the Nyquist plot, this R-CPE element is not visualized in the fit. In all cases, equivalent circuit fits are depicted as a visual guide for the distinction

of processes, supporting the conclusions drawn in the main manuscript, where impedance spectra are discussed qualitatively rather than quantitatively.

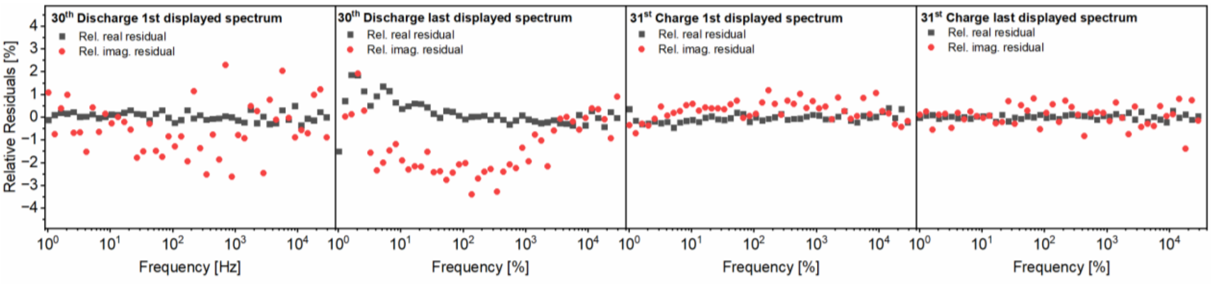

Supplementary Figure 24. **Kramers-Kronig test for DEIS of Cu||NMC622 in coin cells upon the 30th discharge and 31st charge cycle.** Frequency range: 30 kHz – 1.0 Hz. Electrolyte: LHCE (LiFSI:DME:TTE = 1:1.2:3 *n*:*n*:*n*).

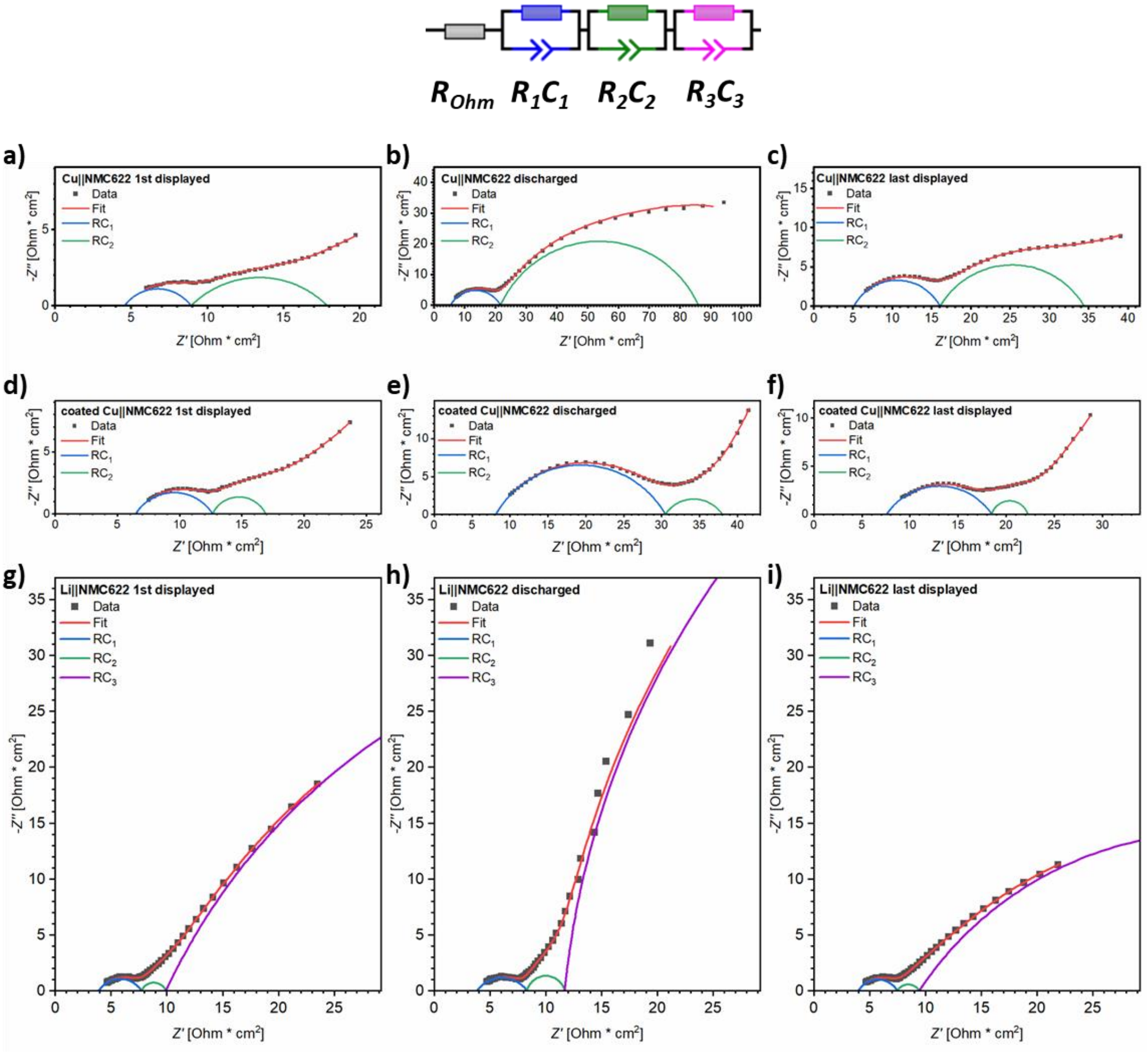

Supplementary Figure 25. **Equivalent circuit fit of the DEIS spectra being 1st displayed, completely discharged and last displayed in the main manuscript. a, d** and **g** Cu||NMC622 coin cells. **b, e** and **h** coated Cu||NMC622 coin cells **c, f** and **i** Li||NMC622 coin cells. Frequency range: 30 kHz – 1.0 Hz. Electrolyte: LHCE (LiFSI:DME:TTE = 1:1.2:3 *n*:*n*:*n*).

Similar to **Supplementary Fig. 17** and **23,** some equivalent circuit fits (i.e. **Supplementary Fig. 25g-i**) are of reasonable uncertainty, since exclusion of data points in the high (> 30 kHz) and particularly

low (< 1 Hz) frequency regime according to Kramers-Kronig tests renders some of the semi-circles incomplete. Furthermore, a third R-CPE element has been implemented to improve fitting of EIS spectra, since a third semi-circle occurs at the low frequency limit. Depending on the number of data points for the third semi-circle and scaling of the Nyquist plot, this R-CPE element is only visualized in **Supplementary Fig. 25 g-i**. In all cases, equivalent circuit fits are depicted as a visual guide for the distinction of processes, supporting the conclusions drawn in the main manuscript, where impedance spectra are discussed qualitatively rather than quantitatively.

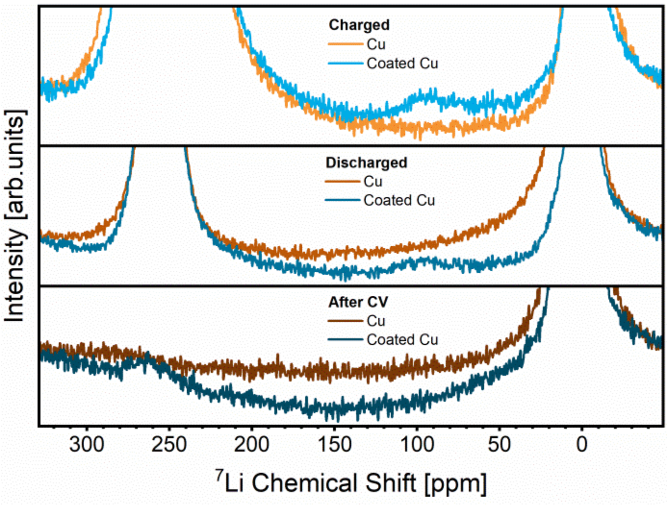

Supplementary Figure 26. **In situ ^{7}Li NMR spectra at regular magnetic field strength (B_0 = 4.7 T) optimized for a detection of Li alloys.** Experiments were conducted in the initial cycle of Cu||NMC622 and coated Cu||NMC622 NMR pouch cells in the charged or discharged state and after the constant voltage discharge step. Spectra are processed with a 0th and 1st order phase correction. Electrolyte: LHCE (LiFSI:DME:TTE = 1:1.2:3 *n*:*n*:*n*).

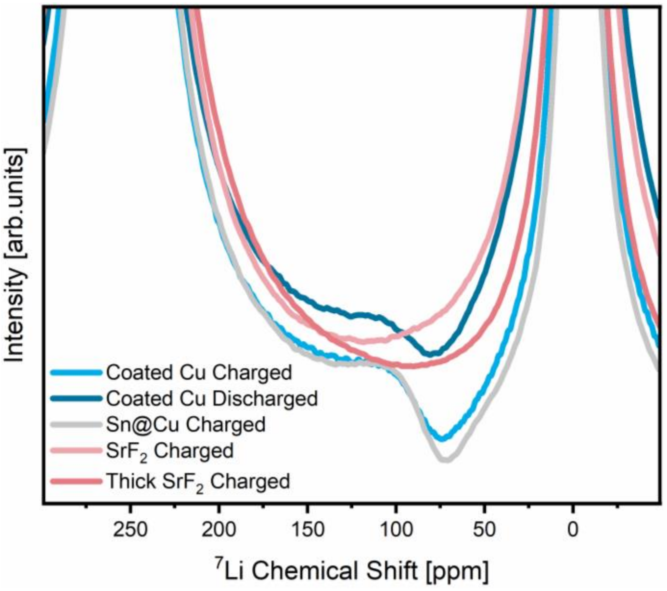

Supplementary Figure 27. **Ex situ ^{7}Li NMR spectra at increased magnetic field strength (B_0 = 14.1 T) for detection of alloy species.** Coated Cu, SrF_2@Cu and Sn@Cu negative electrodes were harvested from NMC622-based anode free full cells. Spectra are processed in magnitude mode. Electrolyte: LHCE (LiFSI:DME:TTE = 1:1.2:3 *n*:*n*:*n*).

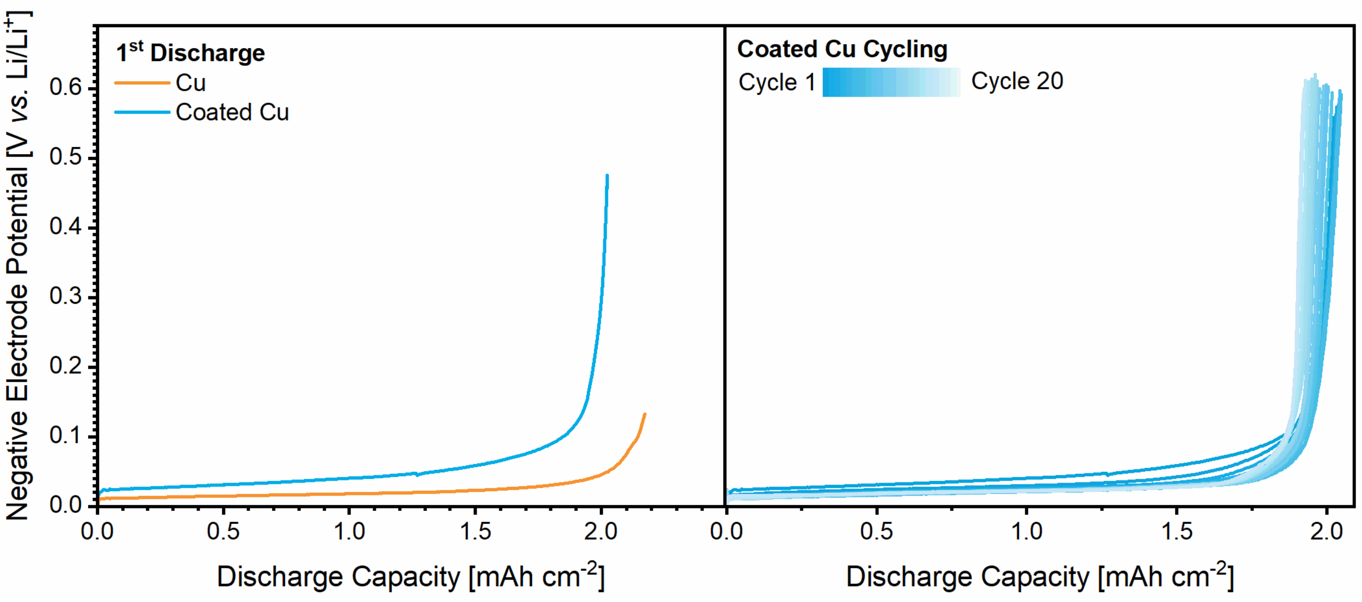

Supplementary Figure 28. **Negative electrode potential profile in Cu||NMC622 and coated Cu||NMC622 three-electrode PAT cells during discharge.** Electrolyte: LHCE (LiFSI:DME:TTE = 1:1.2:3 *n*:*n*:*n*).

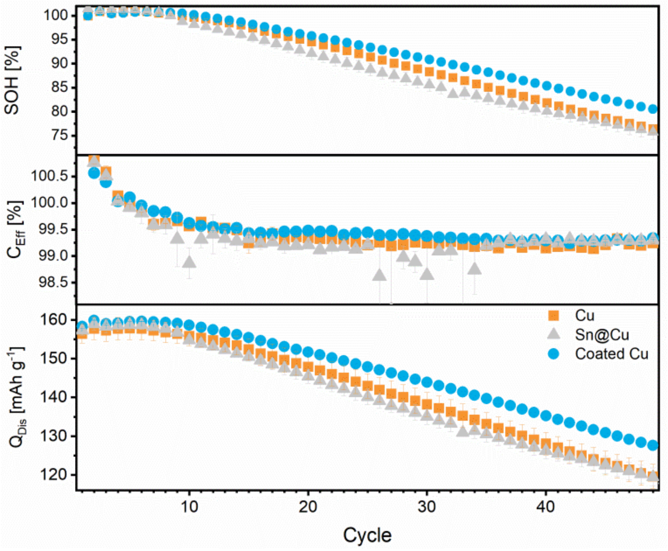

Supplementary Figure 29. **Comparison of discharge capacity (Q_{Dis}), Coulombic efficiency (C_{Eff}) and state of health (SOH) for Cu||NMC622, Sn@Cu||NMC622 and coated Cu||NMC622 coin cells.** Electrolyte: LHCE (LiFSI:DME:TTE = 1:1.2:3 *n*:*n*:*n*). Error bars represent the standard deviation between at least two nominally identical cells.

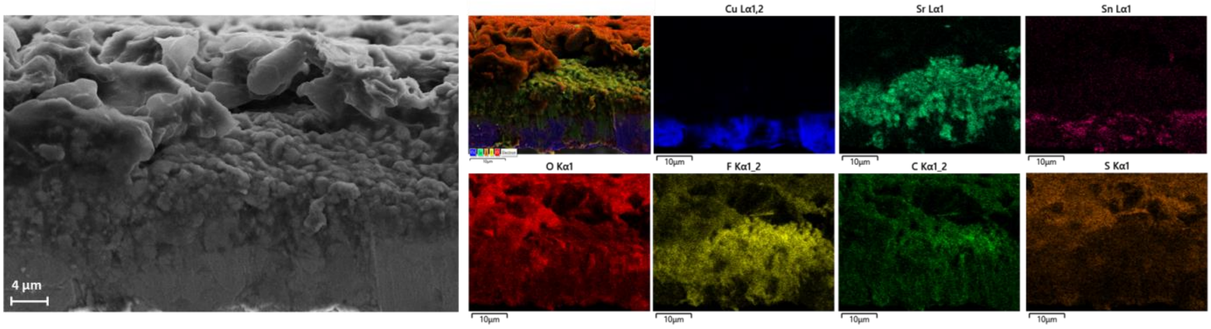

Supplementary Figure 30. **Cross-sectional SEM image and EDX mapping of coated Cu negative electrode harvested from coated Cu||NMC622 coin cells after the initial discharge.** Accelerating voltage: 10 kV. Electrolyte: LHCE (LiFSI:DME:TTE = 1:1.2:3 *n*:*n*:*n*).

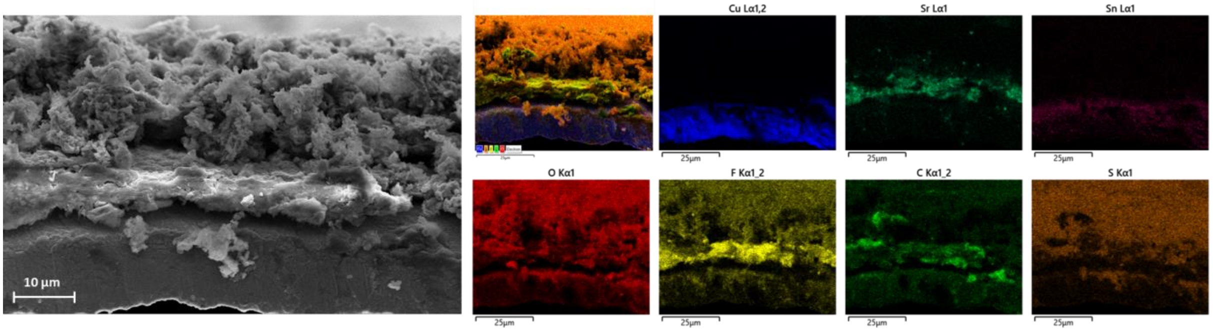

Supplementary Figure 31. **Cross-sectional SEM image and EDX mapping of coated Cu negative electrode harvested from coated Cu||NMC622 coin cells after the 30th cycle in the discharged state.** Accelerating voltage: 10 kV. Electrolyte: LHCE (LiFSI:DME:TTE = 1:1.2:3 *n*:*n*:*n*).

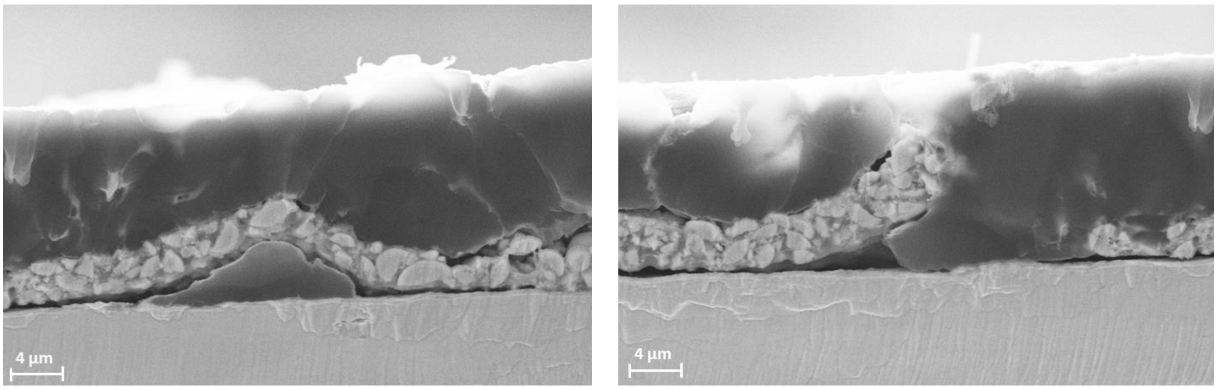

Supplementary Figure 32. **Cross-sectional SEM image of coated Cu negative electrode harvested from coated Cu||NMC622 coin cells after the initial charge to 100% SOC.** Accelerating voltage: 10 kV. Electrolyte: LHCE (LiFSI:DME:TTE = 1:1.2:3 *n*:*n*:*n*).

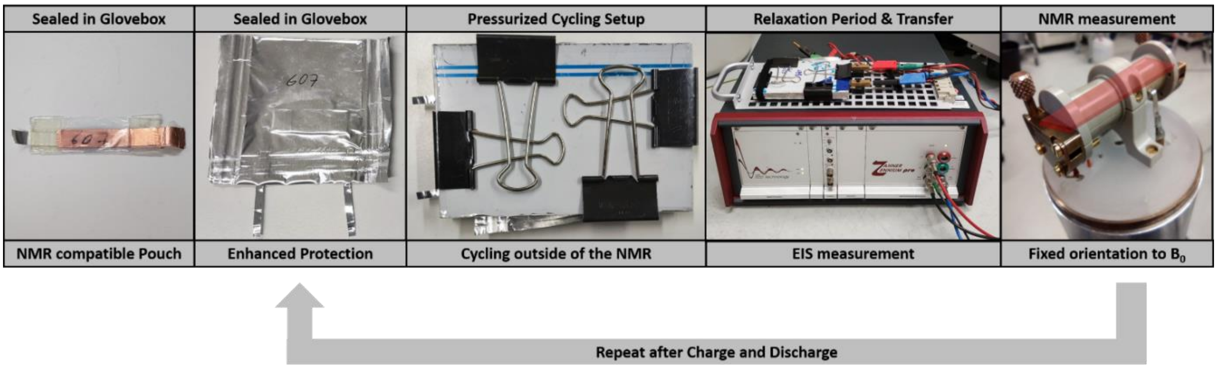

Supplementary Figure 33. **Preparation, operation and EIS/NMR measurements of NMR compatible pouch cells.**

Supplementary References

(1) Shuaibu, A. D.; Mirghni, A. A.; Shah, S. S.; Hardianto, Y. P.; Alzahrani, A. S.; Aziz, M. A. Enhancing Temperature-Optimized Ionic Liquid Electrolytes for High-Voltage, High-Energy Supercapacitors Utilizing Date Stone-Derived Carbon in Coin Cell Configuration. *Battery Energy* **2025.** DOI: 10.1002/bte2.70005.

(2) EL-CELL. *The PAT Core Concept*:*Enabling battery studies of unmatched quality*. https://www.el-cell.com/pat-series/the-pat-core-concept/.

(3) Klink, S.; Madej, E.; Ventosa, E.; Lindner, A.; Schuhmann, W.; La Mantia, F. The importance of cell geometry for electrochemical impedance spectroscopy in three-electrode lithium ion battery test cells. *Electrochemistry Communications* **2012**, *22*, 120–123. DOI: 10.1016/j.elecom.2012.06.010.
